# Surviving indoor heat stress in United States: A comprehensive review exploring the impact of overheating on the thermal comfort, health, and social economic factors of occupants

**DOI:** 10.1016/j.heliyon.2024.e25801

**Published:** 2024-02-03

**Authors:** Chima Cyril Hampo, Leah H. Schinasi, Simi Hoque

**Affiliations:** a Department of Civil, Architectural, and Environmental Engineering, Drexel University, USA; b Department of Environmental and Occupational Health, Drexel Dornsife School of Public Health, Philadelphia, USA

**Keywords:** Indoor overheating, Extreme heat, Thermal comfort, Socioeconomics, Residential buildings

## Abstract

In the face of escalating global climate change and the increasing frequency of extreme heat events, the mitigation of building overheating has become an urgent priority. This comprehensive review converges insights from building science and public health domains to offer a thorough understanding of the multifaceted impacts of indoor overheating on occupants. The paper addresses a significant research gap by offering a holistic exploration of indoor overheating of residential buildings and its consequences, with a specific focus on the United States, an economically diverse nation that has been underrepresented in the literature. The review illuminates the effects of overheating on thermal comfort, health, and socio-economic aspects within the built environment. It emphasizes associated repercussions, including heightened cooling energy consumption, increased peak electricity demand, and elevated vulnerability, leading to exacerbated heat-related mortality and morbidity rates, especially among disadvantaged groups. The study concludes that vulnerabilities to these impacts are intricately tied to regional climatic conditions, highlighting the inadequacy of a one-size-fits-all approach. Tailored interventions for each climate zone are deemed necessary, considering the consistent occurrence of indoor temperatures surpassing outdoor levels, known as superheating, which poses distinct challenges. The research underscores the urgency of addressing indoor overheating as a critical facet of public health, acknowledging direct socioeconomic repercussions. It advocates for further research to inform comprehensive policies that safeguard public health across diverse indoor environments.

## Highlights

• Indoor overheating intensifies due to global climate change, affecting various aspects of occupant well-being.
• Adaptive thermal comfort model preferred in studies, offering a comprehensive approach to understanding occupant responses.
• Vulnerable populations face increased health risks and socioeconomic impacts due to indoor overheating.
• Disproportionate effects of indoor overheating on marginalized communities highlight the need for targeted interventions.

## 1 Introduction

Global climate change intensifies the frequency, intensity, and duration of extreme heat events, [1].These events may lead to indoor overheating, which the Chartered Institution of Building Services Engineers (CIBSE) defines as a situation where the temperature within a building exceeds a comfortable level for a significant period of time, resulting in thermal discomfort, reduced productivity, and potential health risks. In addition to occupant thermal comfort and health, indoor overheating may contribute to increases in energy consumption. All of these impacts may be critical for vulnerable populations, such as older and low-income households [2], who may lack the physiologic or behavioral capacity to adapt [[3], [4], [5]].

Studies have projected that longer and more severe heat waves are expected as the climate continues to warm [6]. The California Environmental Protection Agency forecasts a dramatic rise in the number of extreme heat days affecting both city and countryside inhabitants across California. On average, these areas are predicted to undergo 40–53 days of extreme heat annually by the year 2050, escalating further to an average of 40–99 days per year by the end of the century in 2099. This represents a significant increase from the historical yearly average of just 4 extreme heat days [7]. Wu and Zhou [8] estimate that the increased frequency of heat waves may cause heat wave deaths in the Eastern U.S. to increase 10-fold by 2057–2059, with a projection of between 1400 and 3600 deaths per year by 2058. Several other studies have also highlighted a strong correlation between heat and mortality/morbidity outcomes [[9], [10], [11]]. Moreover, research findings suggest that many and perhaps most heat-related deaths occur in older populations, many of whom spend the majority of their time indoors, and at home, and who may be unable to afford air conditioning [4,7,12].

The impact of indoor overheating emerges as a multifaceted challenge that progressively attracts scholarly attention. A growing body of recent review studies illuminates the complexities underpinning this issue, drawing attention to the confluence of contributing factors, which see a marked escalation over time. Notably, contemporary construction practices, particularly those employing highly insulated materials, are increasingly susceptible to periodic overheating episodes in the context of present climate dynamics [13].

Simultaneously, research findings underscore the evident correlation between urban overheating and the significant surge in cooling energy consumption, the peak demand for electricity, and the consequential escalation in mortality and morbidity rates. Urban overheating also exacerbates local vulnerability, adding another layer of complexity to the issue [14].

Furthermore, the implications of indoor overheating extend beyond the confines of thermal discomfort, permeating deeper into the realm of public health. A surge in evidence indicates an intensification of health risks in residential spaces, attributing this to the amplification of heat exposure, incidents of flooding, and increased chemical and biological contamination. These issues become particularly acute in the face of anticipated climate change scenarios [15,16].

While various studies explore the efficacy of different passive cooling strategies and discomfort indices, there remains an overlooked aspect regarding how these variables intersect with socio-economic factors [17,18]. Moreover, the analysis of urban overheating's influence on health, energy, and the economy is prevalent in current literature. However, its impact on the quality of life of occupants, especially among vulnerable populations, is frequently underrepresented [19].

A synthesis of these studies offers a valuable foundation to understanding overheating and its impacts. Still, it also reveals the limitations of current research. Most reviews are constrained by their geographical focus (primarily the UK context) or singular impact analysis, leaving a significant research gap that warrants further exploration.

Against this backdrop, the aim of this paper is to bridge this identified research gap by offering an all-encompassing understanding of indoor overheating. This comprehensive review will navigate through the multidimensional impacts of overheating, focusing on thermal comfort, health, and socio-economic factors, while taking into account different geographical contexts and demographic profiles. Our research places a special emphasis on the experiences and responses of occupants in the US. This focus is not just relevant due to the underrepresentation of such a diverse and developed economy like the US in current research, but also because the US shares similar economic and climatic condition with many countries globally, making our findings transferrable. Therefore, insights derived from the US context can provide a valuable understanding of indoor overheating impacts and possible mitigation strategies on a larger, global scale. In line with these objectives, the research questions are as follows.

1. What are the primary drivers of indoor overheating in the context of different US climatic conditions and building typologies?
2. How do these drivers impact thermal comfort, energy demand, health, and productivity, particularly for vulnerable populations?
3. How can we critique and refine the current thermal comfort model to better represent real-world conditions and experiences, and apply the identified neutral thermal comfort thresholds to mitigate the effects of indoor overheating?

This review also bridges the professional gap between the fields of building science and public health sciences, which often exist in silos with limited interaction or communication. The paper is organized as follows. First, we present a review of the key concepts related to indoor overheating, including definitions for thermal comfort and an overview of thermal comfort models as well as standardized metrics for thermal comfort, which are drawn from the fields of building science and architectural engineering. Next, we review the impacts of indoor overheating on thermal comfort, health, cost of living, productivity, and cognitive ability. We focus our attention on the impacts of indoor overheating on marginalized communities living in the U.S. The paper concludes with a discussion of the research gaps, mitigating techniques, limitations and recommendations for future research and policy development.

## 2 Methodology

**Fig. 1:**
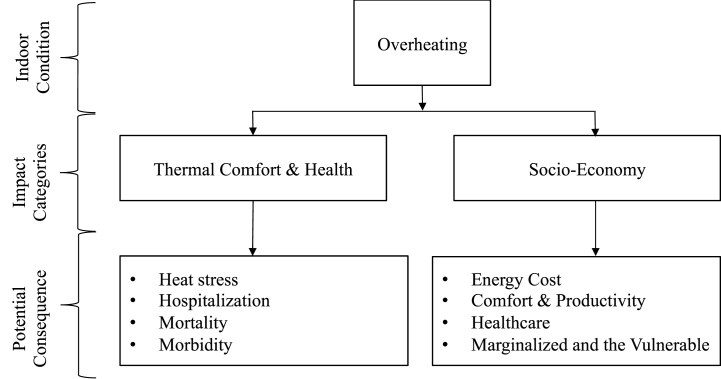
Impact categories and potential consequence of indoor overheating in buildings.

In this review, we captured and synthesized literature that described the impacts of indoor overheating in two broad categories: (1) thermal comfort and health, and (2) socio-economy. Fig. 1 shows the different pathways in which indoor overheating can affect the built environment. This figure was developed based on an illustration patterned by Fisk [20].

### 2.1 Literature search and sorting

To refine our search and retrieve the most relevant research, we employed Boolean operators in Google Scholar. We combined our main topic keywords like "indoor overheating," "heat stress," and "heat waves" with location-specific terms like "buildings" and "homes" using the AND operator to ensure both elements were present in each result. Similarly, we connected specific health outcomes like "asthma," "hospitalization," “morbidity”, and "mortality" using the OR operator to capture studies focused on any of these impacts. For instance, searching for "indoor overheating OR heat stress AND morbidity" would retrieve articles discussing either overheating or heat stress in relation to illness. We further utilized the NOT operator to exclude irrelevant studies by eliminating terms like "outdoor" or "workplace" when focusing on the specific context of indoor environments in residences. Additionally, we incorporated terms like "peak electricity" and "economy" alongside health outcomes to explore the broader consequences of indoor overheating, including its potential impact on energy consumption and societal costs. By strategically applying these Boolean operators, we ensured a comprehensive and targeted search through the vast literature on indoor overheating and its health implications within the United States context. Our search protocol, conducted between November 20, 2022, and April 23, 2023, was broad with no restrictions on publication date, study quality, or study type, given the limited research in this area. However, strict inclusion and exclusion criteria were employed to ensure a focused review.

### 2.2 Inclusion and exclusion criteria

Given the broad scope of our research, the inclusion and exclusion criteria were meticulously established to ensure relevancy and coherence. The literature search was intended to retrieve articles that thoroughly addressed the impacts of indoor overheating on thermal comfort and health, and socio-economic factors. This set the path for the development of our primary inclusion criteria.

Inclusion Criteria:

Papers that were included in the review fulfilled the following criteria.

1. Empirically examined the effects of indoor overheating, focusing on either or both of the categories (thermal comfort and health, and socio-economy).
2. Explored associations with human physiological, psychological, and behavioral responses to indoor overheating.
3. Conducted in the context of residential buildings in the United States.
4. Were either observational or experimental in nature, providing substantive data regarding indoor overheating and its impacts.
5. Published in English.

Exclusion Criteria:

While being inclusive, it was also necessary to establish a comprehensive set of exclusion criteria to maintain the specificity of our review. Papers were excluded from the review if they fell under the following categories.

1. Commentary, editorial, review, or meta-analysis papers were excluded as they may lack primary research data.
2. Lack of investigation into the correlation with ambient temperature, considering the crucial relationship between indoor and outdoor temperatures. This is pivotal because variations in outdoor temperature significantly influence heat transfer through building envelopes.
3. Failure to explore relationships with human physiological, psychological, and behavioral conditions associated with indoor overheating.
4. Primarily focused on associations with infectious diseases (such as diarrhea and parasitic diseases) without relevant evaluation of temperature's mortality/morbidity effects.
5. Concentration on impacts in countries other than the United States, maintaining the geographical focus of this review.
6. Non-observational nature, excluding predictive and risk assessment papers that tend to focus on future projections rather than current conditions.
7. Concentration on occupational studies, as the review primarily focuses on residential buildings.

The meticulous application of these inclusion and exclusion criteria ensured the retrieval of pertinent and coherent studies, establishing a robust foundation for comprehensively reviewing the impacts of indoor overheating.

## 3 Results

### 3.1 Papers identified and sorting result

**Fig. 2:**
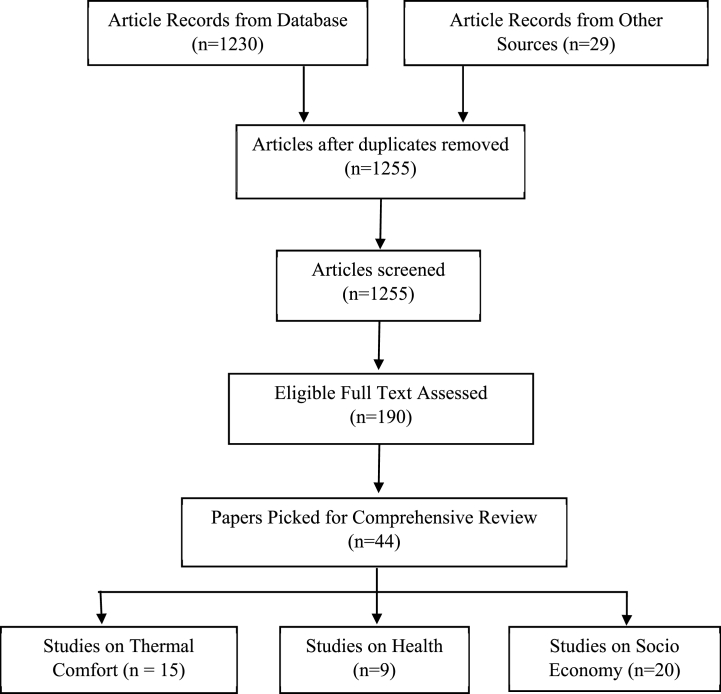
Diagram illustrating the inclusion and exclusion of papers in the comprehensive review.

Conducting an extensive literature review, our automated database search initially yielded 1230 papers, with an additional 29 identified through a meticulous review of reference lists (Fig. 2). Post-duplicate removal, 1255 records persisted. Scrutinizing titles and abstracts resulted in the exclusion of 1069 abstracts, leaving 190 papers for thorough full-text review. Within this corpus, 146 papers were excluded based on distinct criteria, including meta-analytical or commentary nature, absence of ambient temperature association evaluation, lack of mortality/morbidity assessment, failure to explore the overall temperature-mortality association, focus on countries other than the United States, and concentration solely on associations with infectious diseases. Consequently, our comprehensive review encompasses 44 papers, distributed among 15 focusing on thermal comfort, 9 on occupants' health, and 20 on socio-economic aspects.

### 3.2 Thermal comfort

According to the American Society of Heating, Refrigerating, and Air-Conditioning Engineers (ASHRAE), thermal comfort refers to "the state of mind that reflects satisfaction with the thermal environment" and is influenced by environmental factors such as air temperature, humidity, air velocity, and radiant temperature [21]. However, the perception of comfort can vary depending on factors such as age, gender, culture, location, and climate [22]. Different populations or groups may encounter variations in thermal comfort based on various biologic or cultural factors. For instance, challenges in thermoregulation are commonly observed in children and older adults [[23], [24], [25]], with a 50 % of the livings rooms overheated for vulnerable occupants while about 25 % overheated for normal occupants [26].

Poor thermal comfort conditions can have significant negative impacts on health, especially for vulnerable populations like children, older adults, and individuals with underlying illnesses. Exposure to high temperatures and humidity levels can cause heat stress, cardiovascular diseases, respiratory illnesses, and other health issues [27]. To address this, thermal comfort models have been developed to quantify and predict thermal comfort levels under different indoor environmental conditions. These models help to develop guidelines and standards that can maintain optimal thermal comfort levels while minimizing energy consumption. Overall, understanding the importance of thermal comfort and its influence on health is crucial for designing and operating indoor environments that promote occupant comfort, health, and well-being.

#### 3.2.1 Thermal comfort standard models

When assessing the risk of overheating in buildings, it is vital to choose the appropriate thermal comfort model. Two major models that are widely used for thermal comfort in buildings are static and adaptive models.

##### 3.2.1.1 A. static models

Static models, particularly Fanger's PMV (Predicted Mean Vote) and PPD (Predicted Percentage Dissatisfied) models [28], developed in the 1970s, have been commonly incorporated into various comfort standards, such as ASHRAE Standard 55, ISO (International Organization for Standardization) 7730, and EN (European Norms) 15251. These standards employ the PMV/PPD model to define comfort zones, providing system design guidance for various indoor environments. The PMV model integrates six crucial variables to quantify thermal comfort: air temperature, mean radiant temperature, air velocity, humidity, clothing insulation, and metabolic rate. Meanwhile, the PPD model applies the PMV index to estimate the percentage of people in a specific space who would find the thermal conditions unsatisfactory.

However, each model comes with its own strengths and weaknesses. The PMV model's consideration of numerous factors affords a holistic understanding of thermal comfort, but its intricacy requires a vast amount of accurate data and standardizes individual comfort, overlooking personal and cultural variations. Conversely, the PPD model offers a numerical assessment of likely discomfort, making it clear how many people might be unhappy. Despite this, like the PMV model, it requires a lot of inputs and does not clarify the severity of discomfort or if individuals are overly hot or cold. Also, it simplifies the variance in individual thermal comfort. Therefore, while both models provide extensive tools for evaluating thermal comfort, their application in diverse and dynamic environments can be complicated due to these underlying assumptions and extensive data necessities.

##### 3.2.1.2 Adaptive models

The adaptive acceptance model of comfort, which was introduced at the end of the twentieth century, has gained popularity over the years among building professionals, architects, researchers, and policymakers because of its real-world applicability, energy efficiency, resilience, and occupant satisfaction [29,30]. This has led to its incorporation into various building standards and guidelines, such as ASHRAE Standard 55 (2017, 2020), for naturally ventilated buildings and the European standard EN 15251, alongside the traditional static model. The adaptive model acknowledges that the occupants of a building are essential in determining thermal comfort within a building, and that their thermal comfort is influenced by both environmental conditions and their own physiological responses and behaviors [31]. The adaptive model recognizes that different people have different preferences for thermal comfort, which can change based on various factors such as time of day, physical activity, and clothing choices. De Dear and Brager [32] identified three categories of adaptation that occupants use to achieve thermal comfort in indoor environments: physiological, psychological, and behavioral. Physiological adaptation refers to how the human body adjusts to changes in the indoor thermal environment, such as sweating or shivering. Psychological adaptation refers to how the human mind adjusts to changes in the indoor thermal environment, such as adjusting perception based on expectations. Behavioral adaptation refers to how occupants adjust their behavior to achieve thermal comfort, such as adjusting clothing or opening windows. These categories of adaptation can interact, leading to complex responses to changes in the indoor thermal environment.

The static comfort models exhibit effective performance in air-conditioned environments, as reported in Refs. [[33], [34], [35]]. However, these models have a tendency to overestimate discomfort, resulting in technical predictions of increased heating or cooling loads required to achieve thermal comfort [36]. In contrast, adaptive comfort models are suggested for naturally ventilated spaces, especially when occupants have a more direct connection to the outdoor environment [37,38]. Despite the absence of a unanimous consensus regarding the selection of comfort models in mixed-mode buildings, Parkinson et al. [37] demonstrated that these structures align more closely with naturally ventilated buildings in determining neutral temperatures. As a result, the adaptive comfort model is considered more preferable in such scenarios.

De Dear and Brager found that occupants in naturally ventilated buildings are better able to achieve thermal comfort across a wider range of temperatures compared to those in centrally (and statically) controlled HVAC buildings. The adaptive model has been supported by field studies, including ASHRAE-backed studies, which aim to explain the adaptive regression model. This model takes into account various factors such as environmental conditions, occupancy patterns, and building characteristics, to predict the level of thermal comfort desired by occupants at any given time. The use of this model has been shown to improve energy efficiency in buildings while also increasing occupant comfort and satisfaction [30,31,39].

EN (European Norms), ISO (International Organization for Standardization), ASHRAE (American Society of Heating, Refrigerating, and Air-Conditioning Engineers), and CIBSE (Chartered Institution of Building Services Engineers) are all organizations that publish standards for the indoor built environment. These standards provide guidelines and requirements for various aspects of building design, construction, and operation, including thermal comfort. The standards developed by these organizations provide definitions, measurement methods, and criteria for assessing indoor environmental conditions and determining whether they are acceptable for occupants. The adoption of these standards can lead to improved indoor environmental quality, occupant comfort, and energy efficiency in buildings. Therefore, it is important to consider the recommendations of these organizations in achieving thermal comfort in the indoor built environment. The details of the static and adaptive comfort models suggested in EN, ISO, ASHRAE, and CIBSE standards are summarized in Table 1.

**Table 1:** Details of the static and adaptive comfort models as suggested in EN, ISO, ASHRAE, and CIBSE standards.

Origin	Standard	Details	Mechanism	Zone (AS, RH)	Max. Indoor Temp. [⁰C]	Applicable Indoor Temp. [⁰C]
Europe	EN 15251 (2006), EN 16798 (2019)	Set forth by EU Directive 2002/91/EC. Updates on indoor environmental input factors and thermal comfort models	Mechanically heated and cooled	Living Spaces (AS: <0.15 m/s, RH: 30–70 %)	25.5–28	10–30
International	ISO 17772 (2017–2018)	Standards for indoor environmental quality and energy performance assessments	Mechanically heated and cooled	Living Spaces	–	10–30
United States	ASHRAE 55 (2017, 2020)	Specifies thermal conditions for all types of buildings	General	Various (AS: <25 m/s, RH: 20–60 %)	–	10–33.5
United Kingdom	CIBSE Guide A (2006, 2015), CIBSE TM52 (2013), CIBSE TM59 (2017)	Guidelines for HVAC systems, overheating criteria, and evaluating overheating risks	Air-conditioned, Free running, Mechanically and naturally conditioned	Various (AS: <0.15 m/s, RH: 40–60 %)	23 to 28 (+1 °C)	10–30
Germany	Passive House	Voluntary standard for energy efficiency and indoor environmental quality	Without active cooling or with passive cooling	–	–	–

1 The purpose of these standards^2^ is to provide comprehensive guidelines for achieving indoor thermal comfort, taking into account both established best practices and the necessity for adaptability and flexibility in real-world scenarios. Achieving an appropriate balance between adherence to established guidelines and accommodating individual and contextual factors is crucial for effectively managing indoor thermal comfort. It is important to acknowledge that thermal adaptation can be achieved through multiple mechanisms. Physiological adaptation involves the body's physiological response to temperature changes, while psychological adaptation is influenced by past experiences and mental states. Behavioral adaptation entails modifying behaviors in response to thermal conditions [40]. The availability of opportunities for individuals to adapt is pivotal in attaining comfort. Notably, the concept of a comfortable temperature is not static but rather subject to fluctuations [30,41].

It is however worthy of note to mention that the specific details regarding the sample size selection used in defining these thermal comfort models and standards are beyond the scope of our current research focus. Our primary aim is to comprehend and implement the broader guidelines and principles related to managing indoor thermal comfort. Nonetheless, acknowledging the significance of sample size selection in establishing robust standards contributes to a comprehensive understanding of the subject matter.

#### 3.2.2 Effect of extreme heat events on indoor thermal comfort

The increase in extreme heat events presents a significant challenge for indoor thermal comfort, especially in densely populated urban environments where most people spend their time indoors. This issue is particularly pertinent for vulnerable groups such as the elderly and low-income households, where substandard living conditions may already exist.

The table below (Table 2) gives an overview of key studies conducted on this topic. These studies, carried out in various regions across the United States, apply a range of methodologies. They use whole-building energy simulations, environmental and behavioural monitoring, temperature and humidity measurements, and a comparative analysis of indoor and outdoor weather conditions. They aim to understand the correlation between indoor and outdoor temperatures, the impact of building characteristics and construction practices on indoor thermal comfort, and the role of adaptive actions such as air conditioning use and window opening. The insights garnered from these studies play a vital role in forming strategies to enhance indoor thermal comfort and resilience during increased heat events.

**Table 2:** Studies on the effect of extreme heat on indoor thermal comfort.

Ref.	Sample Size/Location	Main Findings	Methods	Thermal Comfort Assessment	Factors Affecting Indoor Conditions
[42]	20 largest metropolitan areas in the US	Significant vulnerability to heat disasters with more than 50 million citizens at risk. Intensification of urban heat islands and climate change worsens the situation.	Whole-building energy simulations	Buildings exceed overheating threshold in 5–7 h on average in half of the simulated locations.	Construction practices, urban development, presence of AC, climate change
[43]	24 senior apartments, 3 public housing sites in NJ, USA	High exposures to overheating during summer. Indoor Heat Index exceeded thresholds of 27 °C. Overheating was higher in the 30s′ low-rise site.	Environmental and behavioural monitoring and interviews	Overheating and poor air quality, even with window opening.	Building age and presence of natural ventilation.
[44]	327 dwellings in New York City, NY, USA	Indoor temperatures are positively and strongly associated with outdoor temperature during the warm season. Apartment buildings and dwellings on higher floors are significantly warmer.	Temperature and humidity measurements	Not specifically addressed, but dwellings in higher socioeconomic status neighborhoods are significantly cooler.	Outdoor temperature, number of rooms in the dwelling, water damage, housing type and location
[45]	16 homes in Greater Boston, MA, USA	Correlation between indoor and outdoor temperatures only at warmer outdoor temperatures. Absolute humidity exhibited the strongest indoor-to-outdoor correlation.	Comparison of indoor and outdoor weather measurements	Not specifically addressed.	Outdoor temperature, apparent temperature, relative humidity, absolute humidity
[46]	145 homes in low-income households in Baltimore city	Large variation in indoor temperatures, with daily-mean indoor temperatures varying from 10 °C lower to 10 °C higher than outdoor temperatures. Houses with central AC are generally cooler than outdoors.	Temperature measurements	Not specifically addressed but noted large variability in indoor and outdoor temperatures.	Availability of AC, outdoor temperature
[47]	30 different homes in Detroit, MI.	Average maximum indoor temperature was 13.8 °C higher than average maximum outdoor temperature.	Hourly indoor temperature measurements, outdoor weather data	Indoor temperatures exceed the comfort range among elderly occupants.	Outdoor temperatures, housing and environmental characteristics
[48]	30 residences in northern Manhattan, NY	Indoor temperatures are far more stable than outdoor temperatures, with the indoor diurnal average typically above the outdoor average.	Sensor measurements, interviews, personal stories	Not specifically addressed.	Building interior thermal inertia

Taken together, these studies underscore the significant impact of extreme heat events on indoor thermal comfort, especially for vulnerable populations such as the elderly and those residing in low-income households. They reveal that indoor temperatures can substantially exceed outdoor temperatures during heatwaves, often beyond comfort range. Factors such as building characteristics, construction practices, air conditioning availability, and adaptive actions of residents play crucial roles in modulating indoor heat stress.

The research suggests that the linear correlation between indoor and outdoor temperatures is significant only at warmer outdoor temperatures, implying that the outdoor temperature may not be an accurate predictor of indoor conditions. Building structures and materials, socioeconomic factors, and floor levels also have a profound impact on indoor temperature variances.

Crucially, the studies highlight a critical need for enhanced building codes and urban planning strategies to consider thermal resilience in addition to energy efficiency. This includes optimizing construction practices, enhancing access to air conditioning, and promoting effective adaptive behaviors. Furthermore, these studies also reinforce the importance of absolute humidity as an effective measure for assessing indoor thermal conditions and personal exposure to heat.

Ultimately, addressing indoor heat stress issues necessitates an integrated approach that combines adaptive strategies, better building design, improved living conditions, and effective climate change mitigation efforts.

### 3.3 Impact of indoor overheating on occupant health

The World Health Organization has clearly articulated that high temperatures can significantly impact human health, leading to an increased rate of premature mortality [49,50]. A variety of health complications can arise from exposure to high ambient temperatures, including cerebrovascular disorders, cardiovascular and respiratory issues, decreased blood viscosity, an increased risk of thrombosis, and impaired kidney function [51].

A report by the Centers for Disease Control and Prevention [12] highlights that, on average, more deaths in the United States result from extreme heat than from any other type of extreme weather event. Those most at risk for hyperthermia death include older adults, individuals with underlying physical or mental health conditions, and those without access to functioning home air conditioning. Between 2000 and 2011, New York City recorded an average of 447 heat-related emergency department visits, 152 hospital admissions, and 13 heat-related deaths annually. Upon examination of medical examiner records for 48 decedents, it was found that 85 % were exposed to heat at home and none of the decedents had a functioning air conditioner in the records where such information was available. Among decedents aged 18–64 years, 48 % were obese and another 29 % were overweight.

While there are many studies investigating the impact of high temperatures on human health during heat waves and other extreme weather events [[52], [53], [54]], not much is known about the relationship between indoor heat exposure and health. This is a crucial gap in knowledge, especially considering that some of the most heat-vulnerable subpopulations, such as the elderly, young children, and those with poor health, spend approximately 81 % of their time indoors at home [20].

Several studies have established the direct human physiological responses and threshold temperature values to indoor heat stress, such as circulatory and respiratory [55,56], blood pressure [57,58], mental health [59], and cognition, dizziness, and fatigue [60,61]. Details of these studies are presented in Table 3.

**Table 3:** Overview of studies on indoor thermal conditions and health outcomes in the US.

Ref.	Focus	Method	Participants	Main Measurements	Key Findings
[55]	Indoor and outdoor heat exposure on COPD morbidity	Longitudinal cohort study	69 participants with COPD	Home environmental monitoring (temperature, humidity, indoor air pollutants), daily respiratory health assessments, portable spirometry	Indoor heat is associated with worsened COPD symptoms and increased rescue inhaler use. Effect magnified with higher indoor air pollution. Outdoor heat linked to increased symptoms.
[56]	Indoor environments during emergency medical care	Pilot study, case-control design	338 respiratory cases, 291 cardiovascular cases, 471 controls	Portable sensors for indoor temperature and humidity measurements	Older patients in warmer buildings experienced hotter indoor temperatures. Indoor humidity adjusted to outdoor conditions. No significant difference in indoor heat exposure between cardiovascular cases and controls.
[57]	Indoor temperature control and ambulatory blood pressure	Cross-sectional study	101 normotensive subjects	Ambulatory blood pressure monitoring, interviews, environmental and occupational condition measurements	Seasonal variations in blood pressure, higher in winter. Industrial plant air conditioning influenced seasonal variations.
[58]	BP response and aerobic capacity in hot indoor environment	Observational study	26 community-dwelling older women	Assessment of blood pressure response, aerobic capacity at different room temperatures	Older adults showed lower blood pressure and reduced aerobic capacity in hot indoor conditions.
[59]	Indoor air temperature and agitation in nursing home residents with dementia	Longitudinal study	21 nursing home residents with dementia	Cohen-Mansfield Agitation Inventory (CMAI) for assessing agitated behaviors, measurement of indoor average temperatures	Higher temperatures are associated with increased agitation in residents with dementia. Maintaining thermally comfortable environment recommended.
[61]	Air conditioning during heat waves and cognitive function	Prospective observational cohort study	44 university students	Daily self-administered cognition tests (Stroop, ADD), measurement of indoor temperatures	Non-air-conditioned residents had slower reaction times and reduced cognitive throughput during heat waves. U-shaped relationship between cognitive performance and indoor temperature.
[60]	Indoor temperature, perceptions, and health outcomes	Cross-sectional study	40 New York City apartments	Measurement of indoor temperature and humidity, survey data on perceptions, health outcomes (sleep quality, symptoms)	Perceptions of indoor temperature matched measured temperature. Sleep quality negatively impacted by high indoor temperature. Heat illness symptoms associated with perceived temperature.
[62]	Indoor heat exposure and mortality/morbidity in the elderly	Time-stratified case-crossover study	Elderly individuals (≥65 years) in Houston, Texas	Modeling of summer indoor heat exposure at the U.S. Census block group level, mortality and emergency hospital admission data	Short-term changes in indoor heat linked to increased cause-specific mortality and morbidity among the elderly. Stronger associations observed in African Americans.
[12]	Heat illness and deaths in New York City	Longitudinal study	New York City residents and homeless persons with heat illness diagnosis from 2000 through 2010	De-identified electronic patient records, death certificates, hospital data, medical examiner records	Approximately 447 heat-related emergency department visits, 152 hospital admissions, and 13 heat-related deaths occurred each year in New York City. Higher rates of heat illness and death were associated with older age and neighborhood poverty; chronic physical and mental health conditions were prevalent comorbidities in decedents. 85 % were exposed at home and none of the decedents had a working air conditioner.

3The studies presented in this table provide valuable insights into the relationship between indoor environmental factors and various health outcomes. The findings highlight the importance of indoor temperature, humidity, and air pollution in influencing respiratory morbidity, cardiovascular health, obesity, blood pressure levels, cognitive function, and well-being especially in the United States. The studies emphasize the significance of optimizing indoor environmental conditions, especially for vulnerable populations such as individuals with chronic obstructive pulmonary disease (COPD), older adults, nursing home residents with dementia, and those exposed to extreme heat. The results underscore the need for sustainable adaptation measures, improved indoor thermal comfort, and the integration of climate change considerations in building design and public health strategies. These findings contribute to our understanding of the complex interplay between indoor environments and health, aiding in the development of targeted interventions to mitigate the adverse effects and promote healthier indoor living environments.

### 3.4 The impact of indoor overheating on socioeconomic factors

The phenomenon of indoor overheating is not just a physiological challenge, but it also carries far-reaching socioeconomic implications. These impacts are notably felt across the complex and varied landscape of communities in the United States. It is crucial to clarify that while higher temperatures do contribute to health problems associated with heat exposure, they represent only a part of the broader issue. In fact, the role of socioeconomic status is critical, with an outsized impact felt by marginalized social segments [48].

Certain communities in the U.S., particularly those that are marginalized, are facing a disproportionate risk of heat-related illnesses and mortality. The root causes behind this heightened vulnerability are not entirely clear, yet the correlation is undeniable [63]. In a report by the CDC [12], it was revealed that nearly half of all heat wave-related fatalities in New York City from 2000 to 2011 occurred within the African American community.

It is important to note that racial divisions often coincide with unique social environments. This fact has been underscored by research carried out in Phoenix, Arizona [64], and Chicago, Illinois [65], which highlighted the crucial role that social networks play in reducing susceptibility to heat stress.

In considering the response to these challenges, various detrimental outcomes must be acknowledged. These include increased energy costs, a decline in productivity, and mounting healthcare costs. Such impacts have a particularly significant bearing on marginalized communities such as low-income households, the elderly, and those living with pre-existing health conditions. Given these findings, it becomes abundantly clear that the issue of indoor overheating demands urgent attention, and that a comprehensive, multisectoral approach is needed to effectively address it.

#### 3.4.1 Energy bills

Socioeconomics plays a dominant role, especially in marginalized social groups, which affects occupants not only with the high cost of energy bills but with the purchase and installation of Air Conditioners and also high cost of rents [48]. This explains why most marginalized communities are among the lowest users of air conditioners [48,66].

Several studies have documented the relationship between indoor overheating and increased energy bills. For instance, a US study by Barreca et al. [67] found that adopting residential air conditioning can increase average household electricity consumption by 11 %, resulting in a consumer surplus of $5 billion to $10 billion annually. Another study by Ref. [68] showed that households with air conditioning on average spend 35%–42 % more on electricity than those without it.

However, there are solutions to mitigate the impact of indoor overheating on households. A study by Ref. [69] in Colorado reported that energy efficiency upgrades alone can reduce HVAC energy use during peak hours by up to 50 % and the HVAC utility bill by up to $312/year. With a proper home energy management system, an additional daily average daily peak demand can be reduced by up to 0.58 MW or 1.2 kW/home in the higher envelope efficiency homes. These findings suggest that investing in energy-efficient building design is an effective strategy for reducing the economic impact of indoor overheating on households.

Given the evidence from these studies, it is crucial to take action to address the economic burden of indoor overheating on households. Improving energy efficiency and reducing indoor overheating can significantly reduce the financial strain on households and improve the quality of life for residents. Therefore, households, builders, and policymakers should prioritize energy-efficient building design and adoption of energy-saving measures to ensure a comfortable living environment without incurring excessive energy bills.

#### 3.4.2 Comfort and productivity

The relevance of understanding the impact of overheating on the comfort and productivity of building occupants has become increasingly crucial, particularly with the growing trend of working from home post covid-19 pandemic. As more individuals engage in remote work, ensuring optimal productivity in home environments is paramount. Overheating has been identified as a factor that can significantly reduce work performance, leading to absenteeism and dissatisfaction with indoor conditions. Numerous studies have highlighted the adverse effects of extreme heat on worker productivity, with findings suggesting a noteworthy reduction in performance with each degree increase in temperature. For example, a study conducted by the Lawrence Berkeley National Laboratory in California [70] focused on a call center, revealing a 2–3% decline in performance for every 1 °C rise in temperature. This impact was most pronounced on cognitive tasks, especially when temperatures exceeded 25.4 °C. The study considered various factors such as ventilation rates, carbon dioxide concentrations, shift length, staffing levels, time of day, and specific tasks. Understanding these dynamics is essential as it directly correlates with the increasing prevalence of remote work, making it imperative to create conducive home environments for sustained productivity.

This finding aligns with a subsequent study conducted in Boston, Massachusetts [61], which focused on the differential impacts of having AC on cognitive function during heat waves among university students living in both AC and non-AC buildings. The study revealed that students in non-AC buildings experienced significantly lower cognitive function, with mean indoor temperatures in non-AC buildings being significantly higher than those in AC buildings. The cognitive function tests administered daily demonstrated an increased reaction time and reduced throughput during heat events in non-AC residences compared to those in AC residences. Moreover, the effects of indoor temperatures on cognitive function were not linear across all tasks, with some exhibiting a U-shaped curve with linear effects below and above an optimum range of indoor temperature (22°C-23 °C). Despite the limitation that the cognitive tests occurred immediately after waking and thus could not assess whether the effects persisted throughout the day, the study offered strong evidence that the indoor thermal conditions during heat waves could adversely impact cognitive function in otherwise healthy individuals.

These findings therefore underscore the importance of maintaining comfortable indoor temperatures to support occupant health and productivity, especially as the trend of remote work continues to gain more popularity. Investing in cooling infrastructure and energy-efficient building design can help to mitigate the negative impacts of indoor overheating and support worker health and productivity. This can have significant benefits for businesses and the overall economy, ensuring that workers can perform at their best and contribute to economic growth and prosperity.

#### 3.4.3 Healthcare cost

In terms of healthcare, the impact of indoor overheating on residents should not be overlooked, as it can lead to serious financial and health consequences. Extreme heat is linked to about hundreds of deaths annually in the United States, as well as numerous hospitalizations and emergency room visits, with costs that can have a substantial impact on individuals and the healthcare system (Centers for Disease Control and Prevention, 2020). Furthermore, individuals with pre-existing health conditions, such as asthma and heart disease, may experience exacerbations during extreme heat events, leading to increased healthcare costs. A two-week heat event in California 2008, led to a combined 650 mortality cases, 1600 hospitalizations, and more than 16000 excess emergency room visits, resulted in nearly $5.4 billion dollars in health costs [[71], [72], [73]]. A similar surge was also reported in another recent study in South Setauket, New York. The authors found that hospital admissions and emergency room visits due to asthma-related complications increase during extreme heat events [74]. Notably, air conditioning can significantly reduce the risk of heat-related hospitalizations for vulnerable populations, such as the elderly, children, and those with pre-existing medical conditions [75].

Indoor overheating has severe consequences for healthcare and the economy, according to the literature. To ensure the well-being of building occupants and prevent significant healthcare costs, it is critical to mitigate the negative impacts of indoor overheating through investments in cooling infrastructure and energy-efficient building design. However, further research is necessary to understand the effects of indoor overheating on vulnerable populations and to identify effective strategies for addressing this issue.

#### 3.4.4 Marginalized communities and indoor heat vulnerability

In Balbus and Malina [76], vulnerability refers to the increased risk or susceptibility of specific subpopulations to health and social impacts. Vulnerability can be influenced by various factors such as biological sensitivity, socioeconomic status, and geography. Some of the groups classified as vulnerable include children, pregnant women, older adults, impoverished populations, individuals with chronic conditions, mobility and cognitive constraints, outdoor workers, and, as a consequence of climate change, those who live in coastal and low-lying riverine zones.

Indoor overheating can have a particularly detrimental effect on these marginalized communities, as they may face limited access to cooling infrastructure and resources, leading to a higher risk of heat-related health problems [77]. Additionally, members of minoritized or marginalized groups may live in particularly hot neighborhoods or housing that is prone to overheating due to neighborhood disinvestments [[78], [79], [80]]. Therefore, addressing indoor overheating is essential to ensure that all individuals have equitable access to safe and comfortable indoor environments.

Multiple studies have provided evidence of the harmful effects of indoor overheating on marginalized, low-income communities, and vulnerable populations, particularly in the US. Tsoulou et al. [43] found that indoor overheating in public housing sites contributes to adverse impacts on health and well-being, particularly among low-income seniors during the summer months. Similarly, Fletcher et al. [81] discovered that high temperatures during the summer are associated with hospital admissions for renal diseases in low-income areas with limited access to air conditioning and cooling mechanisms. As a result, low-income populations are at a greater risk of developing heat-related illnesses in the absence of proper cooling resources. These findings highlight the need for interventions aimed at ensuring that all individuals, regardless of socioeconomic status, have access to adequate cooling resources to prevent the negative impacts of indoor overheating on health and well-being.

A study of acute myocardial infarction occurrence and mortality in residents of the Worcester metropolitan area in Massachusetts, conducted by Madrigano et al. [82] revealed that temperature fluctuations increase the risk of myocardial infarction and mortality, particularly for low-income individuals living in neighborhoods with lower socioeconomic status. Vulnerable populations are, therefore, disproportionately affected by extreme weather conditions. Finally, Zanobetti et al. [83] established that having underlying conditions, such as cardiovascular and respiratory diseases, increase susceptibility to mortality during extreme weather conditions. Low-income populations and individuals with specific health conditions are at an even greater risk of mortality in extreme temperatures, highlighting the urgent need for targeted interventions and policies to protect these groups.

In summary, the reviewed research studies demonstrate inequitable impacts of indoor overheating on marginalized, low-income communities, and other vulnerable populations. Limited access to proper cooling resources, combined with the urban heat island effect, increases the risk of heat-related illnesses and mortality. These findings emphasize the need for policymakers and public health officials to prioritize addressing the underlying factors contributing to indoor overheating and implementing targeted interventions and policies that protect vulnerable populations from the health risks of extreme heat.

## 4 Discussions and limitations

Due to the increasing frequency of extreme heat events resulting from global climate change, the impact of building overheating on indoor occupants has become an urgent issue. Several studies have shown that high indoor temperatures and humidity levels have serious implications not only for thermal comfort but also for energy demand, heat-related health conditions, and socioeconomic implications. These impacts may be particularly substantial for vulnerable populations such as older adults and low-income households.

Thermal adaptation encompasses various mechanisms, including physiological, psychological, and behavioral adaptations, in response to temperature changes [40]. The ability of individuals to adapt is crucial for achieving comfort, highlighting the importance of providing suitable opportunities for adaptation [30,41]. In the context of thermal comfort in buildings, two primary models have been recognized in the literature: static and adaptive models. Among these, the adaptive model, which acknowledges occupants as integral to the comfort system, is widely favored in research studies. This preference stems from its ability to capture the dynamic nature of human comfort needs and responses. The standards presented in this study offer valuable insights into how different regions approach indoor thermal comfort. Each standard reflects the unique climate, building practices, and energy considerations of its origin. This diversity is a strength, allowing standards to be finely tuned to local conditions. In the metropolitan areas studied, the vulnerability to heat disasters is stark. This vulnerability is not uniform across regions, with variations influenced by local factors such as construction practices, urban development, and the prevalence of air conditioning. The intensification of urban heat islands, more pronounced in densely populated regions, exacerbates the situation. The impacts are not solely a consequence of climate change but are modulated by local characteristics, making the regional context a key determinant.

Meanwhile, the World Health Organization and other researchers have recognized that high temperatures can significantly affect human health, including increased mortality rates [20,49]. Medical research indicates that exposure to high temperatures can lead to various health problems such as cerebrovascular disorders, cardiovascular and respiratory issues, decreased blood viscosity, increased risk of thrombosis, and impaired kidney function, especially in vulnerable populations [[9], [10], [11],^51^]. The vulnerability is heightened among older adults, those with underlying health conditions, and individuals without access to functioning air conditioning, emphasizing the socio-economic and regional dimensions of heat-related health impacts.

Regarding the socioeconomic impacts, indoor overheating has significant effects on energy bills, occupant productivity, health, and marginalized societies. An increase in indoor temperature leads to a rise in energy consumption as households try to cool their homes, resulting in increased energy bills. This finding is consistent with prior research, which has documented a positive relationship between indoor overheating and increased energy bills [84]. This suggests that air conditioning can significantly contribute to the economic burden of indoor overheating on households. Studies also suggest that investing in energy-efficient building design can help mitigate the economic impact of indoor overheating [85].

Indoor overheating can also lead to decreased worker productivity and increased absenteeism [70]. Studies also found that high indoor temperatures can reduce focus, attention, and overall cognitive performance, leading to decreased productivity [61,86]. Indoor overheating has led to serious financial and health consequences for indoor occupant [75].

The reviewed studies also provide compelling evidence that indoor overheating has a significant impact on marginalized communities and vulnerable populations, particularly low-income families, communities of color, and the elderly who may lack access to proper cooling infrastructure and resources [43]. These groups face an increased risk of heat-related health problems, including hospitalization and mortality, due to limited access to air conditioning and cooling mechanisms [9,81]. Moreover, vulnerable populations, particularly low-income individuals living in neighborhoods with lower socioeconomic status, are at a higher risk of mortality and morbidity due to temperature fluctuations [82,83]. These findings reinforce the findings from the reviewed studies, demonstrating the need for targeted interventions to protect vulnerable populations from the health risks of extreme heat.

Despite its contributions, this review has certain limitations. First, by focusing exclusively on English-language literature, it may unintentionally overlook relevant studies published in other languages. Second, the emphasis on the United States might not accurately reflect thermal comfort conditions and related health impacts in other parts of the world. Unique indoor thermal comfort conditions arise from climatic variations in distinct regions, and the responses of inhabitants to these conditions can vary.

Furthermore, recent studies, including those by Sailor et al. [42] and Tamerius et al. [44] have extensively documented the influence of outdoor variability on indoor thermal conditions. However, the literature still lacks enough research concentrating on other environmental factors, such as relative humidity, metabolic rate, air speed, and clothing insulation, which could contribute to indoor thermal stress and affect occupants. This shortfall extends to most epidemiological research, which has primarily measured ambient temperatures without fully considering other environmental and physiological variabilities when examining the connections between building science, occupant health, and socioeconomic impact. This oversight is particularly noteworthy since some studies have reported a weak correlation between outdoor and indoor temperatures under varying weather and seasonal conditions.

To address these gaps, future research needs to integrate the adaptive model into epidemiological studies and investigate the impact of a broader range of environmental factors. This approach may provide valuable insights into the effects of indoor overheating on health outcomes, particularly for vulnerable populations at higher risk of heat-related illnesses. By recognizing the unique indoor thermal comfort conditions and occupant responses in different climatic zones and considering the potential influence of various environmental variables, future research can contribute to the development of more effective mitigation strategies and thermal comfort standards that addresses the complexities of indoor thermal stress.

## 5 Mitigating techniques

The impacts of thermal discomfort on building occupants have been thoroughly documented, and this paper suggests several approaches to mitigate these effects, including the use of adaptive comfort models, energy-efficient buildings, and policies promoting natural ventilation, shading, and insulation materials.

As proposed by Sameni et al. [26], the adaptive thermal comfort model offers a more transparent approach to assessing the risk of indoor overheating. This model considers occupant vulnerability and actual outdoor temperature, contrasting with the static method [87]. Their findings indicate that the adaptive benchmark reports a lower risk of overheating for normal occupants, while vulnerable occupants face higher risks, with approximately 50 % of living rooms overheated for the latter compared to about 25 % for the former.

Additionally, investigations into the impact of retrofitting on overheating risk have been conducted [[88], [89], [90]]. These studies reveal that overheating risk tends to increase with floor level in high-rise structures, with attics experiencing the strongest heat load [89]. Enhanced roof insulation and window retrofitting have been identified as measures that decrease overheating risk [89,91]. High energy-efficient buildings exhibit greater resilience to overheating than older buildings, provided adequate ventilation is maintained. However, without proper ventilation, the risk of overheating in high-energy-efficient buildings can surpass that in older buildings. The results further demonstrate that, under current climate conditions, natural ventilation in high-energy-efficient buildings is effective in reducing overheating risk but may necessitate additional shading under future climates [92].

Policy measures are integral in mitigating the consequences of thermal discomfort on human health. Consideration of both non-physiological factors like geographical location, urban density, and building design, as well as physiological factors such as sex, age, fitness, and health status, is essential in establishing indoor temperature thresholds that safeguard human health amid the warming global climate [93]. Revisions to building codes and standards are suggested to ensure the resilience of new buildings against elevated temperatures resulting from climate change. This involves integrating passive building designs to diminish reliance on energy-intensive cooling systems [93].

Furthermore, our research recommends the provision of financial assistance to vulnerable populations to enable them to afford air conditioning units or access public cooling centers during heat waves. Public education on the health risks associated with elevated indoor temperatures is also crucial for mitigating the effects of indoor thermal stress on human health. Investing in energy-efficient building design not only alleviates financial burdens on households but also enhances their overall quality of life. These initiatives play a vital role in safeguarding vulnerable populations from both health and economic repercussions linked to indoor overheating.

In summary, a multi-pronged approach involving both technical and policy interventions is required to mitigate the impacts of thermal discomfort on occupants of buildings. This approach could involve implementing energy-efficient measures, improving ventilation and air conditioning systems, revising building codes and standards, providing financial support to vulnerable populations, and educating the public about the health risks associated with high indoor temperatures. By implementing these approaches, we can minimize the impacts of thermal discomfort on the health, productivity, and quality of life of building occupants while reducing the overall energy consumption of buildings.

## 6 Conclusion

The standards presented by EN, ISO, ASHRAE, and CIBSE play a crucial role in guiding indoor thermal comfort practices. Notably, EN standards are attuned to the mechanically heated and cooled environments in Europe, while ISO 17772 serves as a global benchmark. ASHRAE 55 specifies thermal conditions for various U.S. buildings, and CIBSE offers diverse guidelines for the UK. The Passive House standard stands out for its emphasis on energy efficiency without active cooling. These standards encompass a range of mechanisms, zones, and temperature specifications to address diverse climatic conditions. However, to enhance these standards, future revisions should consider the following aspects, which includes, global consistency, adaptive mechanisms, behavioral adaptation, dynamic comfort, sample, size considerations. By addressing these considerations, future revisions can further refine and improve existing standards, making them more adaptable, comprehensive, and globally consistent in guiding indoor thermal comfort practices.

As evident, research conducted in these case studies has highlighted specific vulnerabilities tied to regional climatic conditions. Considering the diverse climate zones represented by these cities, ranging from the colder climates of Boston to the warmer climates of Miami and Phoenix, it becomes evident that a one-size-fits-all approach to thermal comfort may not be appropriate. Further research that takes into account the unique climatic characteristic of each region is essential for developing targeted strategies for mitigating indoor overheating. Superheating, as highlighted in these studies, poses significant challenges, especially for vulnerable populations, and necessitates tailored interventions for each climate zone to ensure indoor environments are conducive to human health and well-being. Our conclusion resonates with the insights of Zou et al. [94], who underscored the crucial role of reliable climate data in both understanding the impacts of overheating and devising effective mitigation strategies across present and future climatic conditions.

Given the heterogeneity of health outcomes associated with indoor thermal conditions, it is evident that addressing indoor overheating is not solely a matter of comfort but a vital aspect of public health. The studies collectively advocate for interventions that consider both specific health vulnerabilities and demographic characteristics. Moreover, the prevalence of heat-related health issues, as highlighted in Ref. [12], emphasizes the urgency of developing targeted strategies, especially for vulnerable populations, to mitigate the health impacts of indoor overheating. Further research in this domain is imperative to inform comprehensive policies that safeguard public health across diverse indoor environments.

The socioeconomic impacts of indoor overheating have also been well-established, with studies reporting significant effects on energy bills, occupant productivity, health cost, and marginalized societies. Indoor overheating has been found to lead to increased energy consumption as households try to cool their homes, resulting in increased energy bills. However, investing in energy-efficient building design can help mitigate the economic impact of indoor overheating. Indoor overheating can also lead to decreased worker productivity and increased absenteeism, while serious financial and health consequences can be experienced by indoor occupants. The reviewed studies have also provided compelling evidence that indoor overheating has a significant impact on marginalized communities and vulnerable populations, particularly low-income families, communities of color, and rural populations, who may lack access to proper cooling infrastructure and resources. These groups face an increased risk of heat-related health problems, including hospitalization and mortality, due to limited access to air conditioning and cooling mechanisms. This highlights the disproportionate impact of extreme temperatures on low-income populations and individuals with specific health conditions. By focusing on the US context, this study contributes valuable insights for policymakers and practitioners to develop targeted interventions and foster more resilient and sustainable built environments in the face of escalating climate change challenges.

Lastly, the reviewed literature has demonstrated the need for targeted interventions to protect vulnerable populations from the health risks of extreme heat. The exploration of emerging technologies like Artificial Intelligence (AI) and the Internet of Things (IoT) are interesting future research innovations. AI has the potential to devise intelligent control systems capable of optimizing HVAC operations in response to real-time indoor and outdoor conditions. Such systems learn from past data, making anticipatory adjustments to enhance occupant comfort while reducing energy use. This strategy can be particularly beneficial for vulnerable populations by ensuring their living conditions are maintained within safe temperature limits during extreme weather events. Likewise, IoT devices can automate home systems, including air conditioning, heating, and window shades. These devices, responding to real-time data, can make adjustments to optimize energy use and maintain comfortable temperatures. Additionally, more studies incorporating other environmental factors like relative humidity, metabolic rate, air velocity, and clothing factor should be seriously considered by researchers so as to have a broader knowledge of their influence.

## Data availability statement

No data was used for the research described in the article.

## CRediT authorship contribution statement

**Chima Cyril Hampo:** Writing – review & editing, Writing – original draft, Validation, Methodology, Investigation, Formal analysis, Data curation, Conceptualization. **Leah H. Schinasi:** Writing – review & editing, Supervision, Resources, Methodology, Funding acquisition, Conceptualization. **Simi Hoque:** Writing – review & editing, Supervision, Resources, Project administration, Methodology, Conceptualization.

## Declaration of competing interest

The authors declare the following financial interests/personal relationships which may be considered as potential competing interests: Leah H. Schinasi reports financial support was provided by National Heart Lung and Blood Institute. Leah H. Schinasi reports a relationship with National Heart Lung and Blood Institute that includes: funding grants.

The authors declare that they have no known competing financial interests or personal relationships that could have appeared to influence the work reported in this paper.

## Footnotes

1 The numbers and letters in the names of thermal comfort standard models represents the standard identification number and the issuing organization. These identification numbers and issuing organizations help to distinguish and standardize the different thermal comfort models and their guidelines across different regions and countries. For more details on static and adaptive thermal comfort standard models, check [Lomas, K.J. and S.M. Porritt, *Overheating in buildings: lessons from research*. 2017, Taylor & Francis. p. 1–18.].

2 Meaning of the acronyms used in Table 1. AS: Air Speed; RH: Relative Humidity; EU: European Union; EN: European Norm; ISO: International Organization for Standardization; ASHRAE: American Society of Heating, Refrigerating and Air-Conditioning Engineers; CIBSE: Chartered Institution of Building Services Engineers.

3 Meaning of the acronyms used in Table 3. COPD: Chronic Obstructive Pulmonary Disease; BP: Blood Pressure; ADD: Attention Deficit Disorder; CMAI: Cohen-Mansfield Agitation Inventory.
